# Determination of Mycotoxins in Plant-Based Meat Alternatives (PBMAs) and Ingredients after Microwave Cooking

**DOI:** 10.3390/foods13020339

**Published:** 2024-01-21

**Authors:** Francesco Giuseppe Galluzzo, Gaetano Cammilleri, Andrea Pulvirenti, Erika Mannino, Licia Pantano, Vittorio Calabrese, Maria Drussilla Buscemi, Elisa Maria Domenica Messina, Calogero Alfano, Andrea Macaluso, Vincenzo Ferrantelli

**Affiliations:** 1 Istituto Zooprofilattico Sperimentale della Sicilia “A. Mirri”, 90129 Palermo, Italy; gaetano.cammilleri@izssicilia.it (G.C.); manninoerika@gmail.com (E.M.); drussilla.buscemi@izssicilia.it (M.D.B.); elisa.messina@izssicilia.it (E.M.D.M.); calogero.alfano@izssicilia.it (C.A.); andrea.macaluso@izssicilia.it (A.M.); vincenzo.ferrantelli@izssicilia.it (V.F.); 2 Dipartimento Scienze della Vita, Università Degli Studi di Modena e Reggio Emilia, 41125 Modena, Italy; andrea.pulvirenti@unimore.it; 3 Dipartimento di Scienze Biomediche e Biotecnologiche, Università degli studi di Catania, 95123 Catania, Italy; calabres@unict.it

**Keywords:** mycotoxin degradation, mycotoxins, PBMAs, microwave, mass spectrometry

## Abstract

In this study, we investigate the role of microwave cooking in reducing mycotoxin contamination in plant-based food matrices, with a focus on veggie burgers (purchased and home-made) and their ingredients (soybean, potatoes, zucchini, carrots). Two different conditions were studied (Max–Min) that were 800 W for 60 s and 800 W for 90 s, respectively. The degradation patterns of aflatoxins (AFB1, AFB2, AFG1, AFG2), fumonisins (FB1, FB2, FB3), trichothecenes (T2, HT2, ZEA), and ochratoxin A (OTA) were studied. The extraction procedures were conducted with the QuEChERS extraction, and the analyses were conducted with liquid chromatography–tandem mass spectrometry (LC-MS/MS). Principal component analysis (PCA) showed that degradation under microwave cooking varies considerably across different food matrices and cooking conditions. This study provides valuable insights into the degradation of mycotoxins during microwave cooking and underscores the need for more research in this area to ensure food safety.

## 1. Introduction

Mycotoxins are secondary metabolites primarily produced by *Aspergillus*, *Fusarium*, and *Penicillium* species and can contaminate a wide range of agricultural products under specific environmental conditions [1,2,3]. These compounds are noted for their toxic effects, including carcinogenesis, immune deficiency, organ damage, teratogenicity, and hormonal imbalances [4,5,6]. Due to these hazardous effects, mycotoxins are regulated worldwide [7]. In the European Union, the maximum levels (MLs) permitted in food matrices are established by Commission Regulation (EU) 2023/915 [8], and the analytical methods must have the performance criteria set by Commission Regulation (EC.) No. 401/2006 [9].

The consumption of plant-based foods and meat alternatives (PBMAs) has increased significantly in recent years due to a convergence of factors related to health, environmental concerns, and ethical considerations [1,10,11,12]. The environmental impact of meat production, particularly in terms of greenhouse gas emissions and animal welfare, is a major factor in the shift towards plant-based eating. It is projected that PBMA products will see further growth in the future, and some studies have projected that the market value of these products will be USD 95 billion by 2030 [13].

Mycotoxin contamination in these products, both pre-harvest and post-harvest, is a prevalent issue due to various factors [12]. Raw ingredients such as cereals (soybeans), which are commonly used in these product types, are particularly susceptible to mycotoxin contamination [14,15,16,17]. The contamination of these substances in PBMAs has been documented by various researchers. Aflatoxins (AFs), fumonisins (FBs), ochratoxin A (OTA), zeranol (ZEA), enniatin, and beauvericin were found in products that are already commercialized in the European market [16,18,19,20]. Mihalache et al. (2023) reported the co-occurrence (84.6%, 11/13) of different mycotoxins in soy burger, soy meat, and soy steak, particularly FBs that were the most frequently detected, followed by tentoxin (TEA), OTA, and AFB1 [18].

Similar results were reported by Carrasco et al. (2019) where the co-occurrence of mycotoxins was 94.4% (17/18) with the presence of up to six mycotoxins in the same soy-based burger [21]. However, to date, these products and some of their most common ingredients (soybeans, peas) are not regulated by European regulations [8,18,22].

Mycotoxins are heat resistant; therefore, their presence in food matrices cannot be eliminated with the most common cooking methods. This characteristic varies significantly depending on the specific type of mycotoxin. AFs are highly stable at high temperatures and can withstand normal food-cooking processes such as boiling and baking [23,24]. FBs are more susceptible to heat treatment, while ZEA, OTA, DON, and T2 are more stable [25]. However, the degradation in the cooking processes depends on the analyzed matrices [25,26,27,28]. The aim of this study is to evaluate the impact of the microwave cooking method on veggie burgers and some of the ingredients that can compose them. Carrots, zucchini, soybeans, and potatoes were used to make a homemade veggie burger that was compared with purchased burgers. Each matrix was fortified and cooked in the conditions reported on the package of the purchased burgers (60 s at 800 W). Furthermore, each matrix was cooked to a “Max” condition that is 50% more than the time labeled on the package (90 s at 800 W). The analysis was conducted with liquid chromatography–tandem mass spectrometry (LC-MS/MS) that allows the analysis of different mycotoxins in different matrices with a single run [16,29,30,31].

## 2. Materials and Methods

### 2.1. Chemicals and Materials

All the solvents used for the analyses were of LC-MS grades (>99.9%). Methanol and acetonitrile were purchased from Sigma-Aldrich (Amsterdam, Holland). The Milli-Q system (Millipore Burlington, MA, USA) was used to obtain ultrapure water.

An aflatoxin mix (AFB1, AFB2, AFG1, AFG2) and fumonisin mix (FB1, FB2, FB3) were purchased from Romer Labs (Getzerdorf, Austria). HT2, T2, OTA, and OTA-d5 were purchased from HPC Standards GmbH (Cunnersdorf, Germany). The extraction and purification procedures were conducted with a Supel QuE Citrate (EN) Tube (55227-U, 4 g MgSO_4_, 1 g NaCl, 0.5 g sodium citrate dibasic sesquihydrate, and 1 g sodium citrate tribasic dihydrate) and Supel QuE P.S.A. (primary, secondary amine, EN) Tube (55228-U, 0.9 g MgSO_4_ and 150 mg of Supelclean P.S.A.).

### 2.2. Sample Collection

The zucchini, carrots, potatoes, soybeans, and purchased soy burgers used in this study were acquired from a retail vendor in Palermo, southern Italy. For uniformity, the carrots, potatoes, and zucchini were sliced using a vegetable-cutting machine. The soybeans were homogenized using a Büchi B-400 vertical mixer (Flawil, Switzerland). Homemade burgers were prepared by combining carrots, potatoes, zucchini, and soybeans, which were first individually prepared and then combined in a homogenized mixture. This preparation ensured a consistent and even blend of ingredients in each burger. All the matrices were stored at −10 °C until analyses. Raw matrices were analyzed, and no mycotoxins were found.

### 2.3. Cooking Procedure

The microwaving parameters were based on the instructions provided on the packaging of the purchased soy burgers, which recommended cooking for 1 min at 800 watts (“Min” condition). An alternative condition tested was cooking for 90 s at 800 watts (“Max” condition). This was the maximum power allowed in the microwave used. These were the only conditions examined, as extending the cooking time resulted in the burning of the hamburgers. Five grams of each food sample was microwaved separately under these conditions. The materials were placed in a 50 mL Falcon tube and cooked with 5 mL of water. The cap was partially open to prevent an explosion or material leakage. The samples were prepared and fortified following the methodology outlined in Pantano et al. (2021) [29]. The initial mycotoxin concentrations in the matrices were as follows: ochratoxin A (OTA) at 3.0 μg/kg, aflatoxins (AFB1 at 1.6 μg/kg, AFG1 at 1.6 μg/kg, AFB2 at 0.4 μg/kg, AFG2 at 0.4 μg/kg), zearalenone (ZEA) at 75 μg/kg, fumonisins (FB1, FB2, and FB3 each at 400 μg/kg), T-2 toxin at 25 μg/kg, HT-2 toxin at 25 μg/kg, and OTA-d5 at 3.0 μg/kg. The spiking procedures were carried out on the surface of the products. Solutions containing the same amounts of mycotoxins were cooked in the same conditions to test the degradation in water. Temperatures were measured immediately after the cooking with a calibrated electronic contact thermometer (IKA™ ETS-D5, Staufen, Germany)

### 2.4. Extraction Procedure and Instrumental Analyses

The instrumental equipment was a Thermo Fisher Ultra High-Performance Liquid Chromatography (UHPLC) system (Thermo Fisher Scientific, California, CA, USA) with of an ACCELA 1250 quaternary pump and an ACCELA autosampler. The column used was a Thermo Scientific Hypersil Gold reversed-phase UHPLC column (50 mm, 2.1 mm ID, 1.9 μm). The chromatography run, the instrumental condition, and the extraction procedure were performed as described previously by Pantano et al. (2021) [29]. Briefly, 5 g of the samples was added to a 50 mL Falcon tube and 10 mL of bidistilled water and 10 mL of an acetonitrile/formic acid (2%) solution were added to the sample. It was vortexed for 15 min and then left to rest at −20 °C for another 15 min. Next, Tube 55227-U was added, followed by shaking for 1 min and centrifugation at 5000 rpm for 10 min. The supernatant was then transferred to Tube 55228-U, shaken for 1 min, and centrifuged for 5 min at 5000 rpm. Finally, 3 mL of the supernatant was evaporated at 40 °C and redissolved in 600 μL of a 50/50 *v*/*v* methanol/water solution, rendering the sample ready for injection. All the analyses were performed in triplicate. Results obtained from the instrument (μg/L) were converted to (μg/Kg) with a conversion factor of 0.4.

### 2.5. Method Performance and Data Accuracy

Linearity was assessed with matrix-matched standard overs the concentrations of 0.8, 2, 4, and 8 μg/L for AFG1 and AFB1, 0.2, 0.5, 1, and 2 μg/L for AFG2 and AFB2; 200, 500, 1000, and 2000 μg/L for FB1, FB2, FB3; 12.5, 31.25, 62.50, and 125 μg/L for T2 and HT2; 1.5, 3.75, 7.50, and 15 μg/L for OTA; and 37.50, 93.75, 187.50, and 375 μg/L for ZEA. Specificity was assessed by analyzing 5 blanks for each matrix and no interferences were found. Forty blank samples were analyzed for each group, *n* = 20 for zucchini and carrots and *n* = 20 for potatoes, soybeans, and purchased hamburger. Limits of detection (LODs) and limits of quantification (LOQs) were determined by identifying the lowest analyte concentrations at which the signal-to-noise ratios equalled 3 and 10, respectively. The linearity was R^2^ > 0.95 for all the analytes. Recoveries were tested by fortifying blank raw samples at the spiking concentration studied. The LOQ-LOQ values for zucchini–carrots (µg/kg) were: AFB1 (0.335–1.105), AFB2 (0.03–0.098), AFG1 (0.367–1.212), AFG2 (0.074–0.243), FB1 (62.922–207.644), FB2 (55.943–184.613), FB3 (67.265–221.974), T2 (6.74–22.26), HT2 (5.66–18.68), OTA (0.43–1.42), ZEA (8.28–27.34). For hamburgers–potatoes–soybeans they were: AFB1 (0.092–0.305), AFB2 (0.012–0.04), AFG1 (0.168–0.555), AFG2 (0.022–0.07), FB1 (17.017–56.15), FB2 (21.77–71.86), FB3 (24.61–81.21), T2 (2.88–9.51), HT2 (2.07–6.83), OTA (0.305–1.008), ZEA (4.33–14.28). Mean recoveries were between 75% and 125% for all the mycotoxins tested in all the matrices.

### 2.6. Statistical Analyses

Samples were named depending on the matrix (Carrots, Zucchini, Soybeans, Potatoes, H. homemade, H. purchased) and grouped by the cooking condition in “Min” and “Max”. All variables were tested for normal distribution using the Shapiro–Wilk test.

The statistical analysis was conducted in two primary phases: the differences between matrices (Carrots, Zucchini, Soybeans, Potatoes, H. homemade, H. purchased) and within matrices (Max–Min). The first was evaluated with the Kruskal–Wallis H test, while the second depended on the normal distribution. When normally distributed, the Welch two sample *t*-test was used to evaluate differences in means between groups (Max–Min) among the same matrices. For variables that did not fulfill the normality distribution assumption, the Mann–Whitney U test was used instead. A principal component analysis (PCA) was conducted after the data were scaled with Pareto scaling [32,33]. A Kaiser–Meyer–Olkin (KMO) test showed an MSA of 0.5, and the *p*-value of Bartlett’s test of sphericity was <0.05. The number of principal components (PCs) to keep was assessed with the Kaiser–Harris criterion, Cattell scree test, and parallel analysis as described in Kabacoff (2021) [34]. Two PCs were kept because they were enough to explain 79.5% of the variance. Statistical analyses were conducted with R 4.1.2 software (freeware available at https://cran.r-project.org/ (accessed on 13 August 2023)). All tests were performed with a 5% significance level.

The percentage of mycotoxins lost in hamburgers was calculated with the following equation:

(1) $$\%Lost=100*\frac{\left(mean\;value\;found\;after\;cooking\right)}{values\;fortified}$$

## 3. Results

### 3.1. Impact of Microwave Cooking on Mycotoxin Degradation

**Figure 1:**
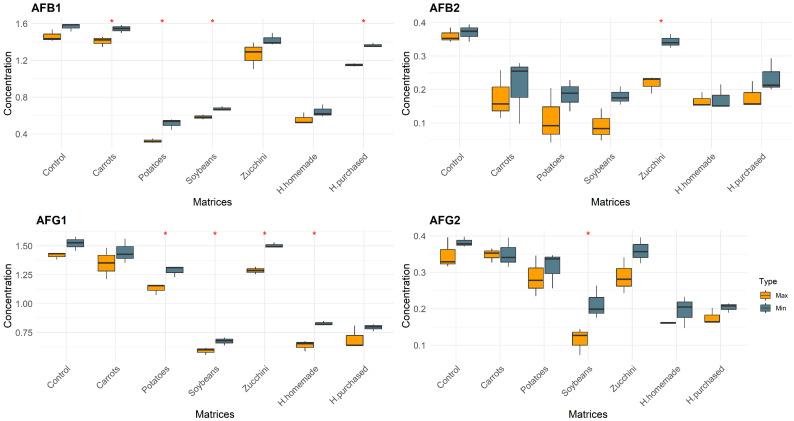
Concentration of AFs after cooking procedure divided by matrices and colored by condition of cooking. “*” represents statistically significant differences (*p* < 0.05).

**Figure 2:**
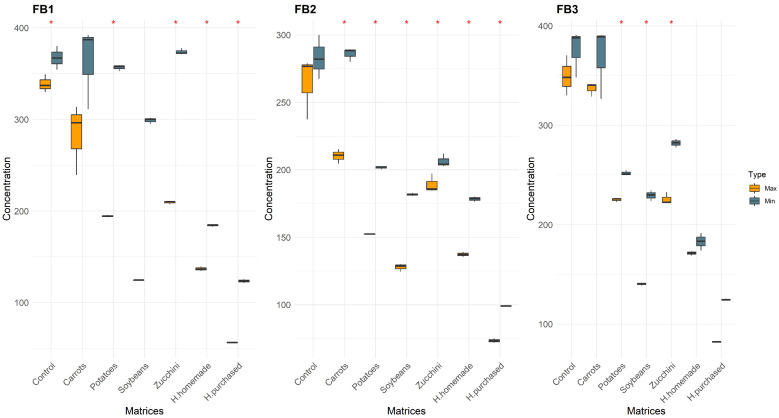
Concentration of FBs after cooking procedure divided by matrices and colored by condition of cooking. “*” represents statistically significant differences (*p* < 0.05).

**Figure 3:**
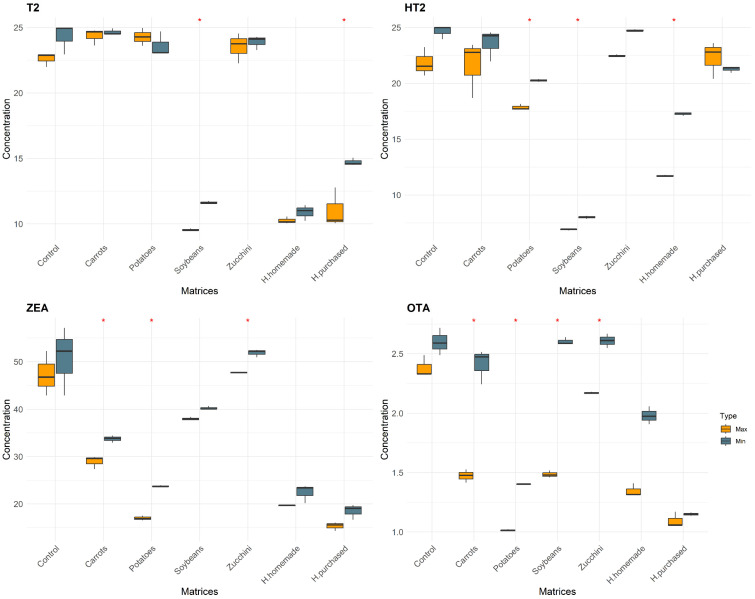
Concentration of T2, HT2, ZEA, and OTA after cooking procedure divided by matrices and colored by condition of cooking. “*” represents statistically significant differences (*p* < 0.05).

**Figure 4:**
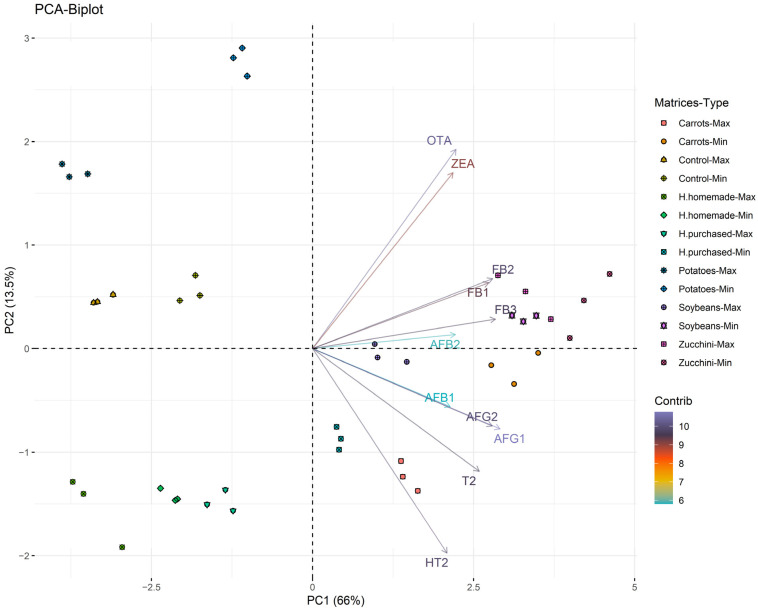
PCA variables are colored depending on the contribution to the PCs (purple->blue in decrescent order).

**Figure 5:**
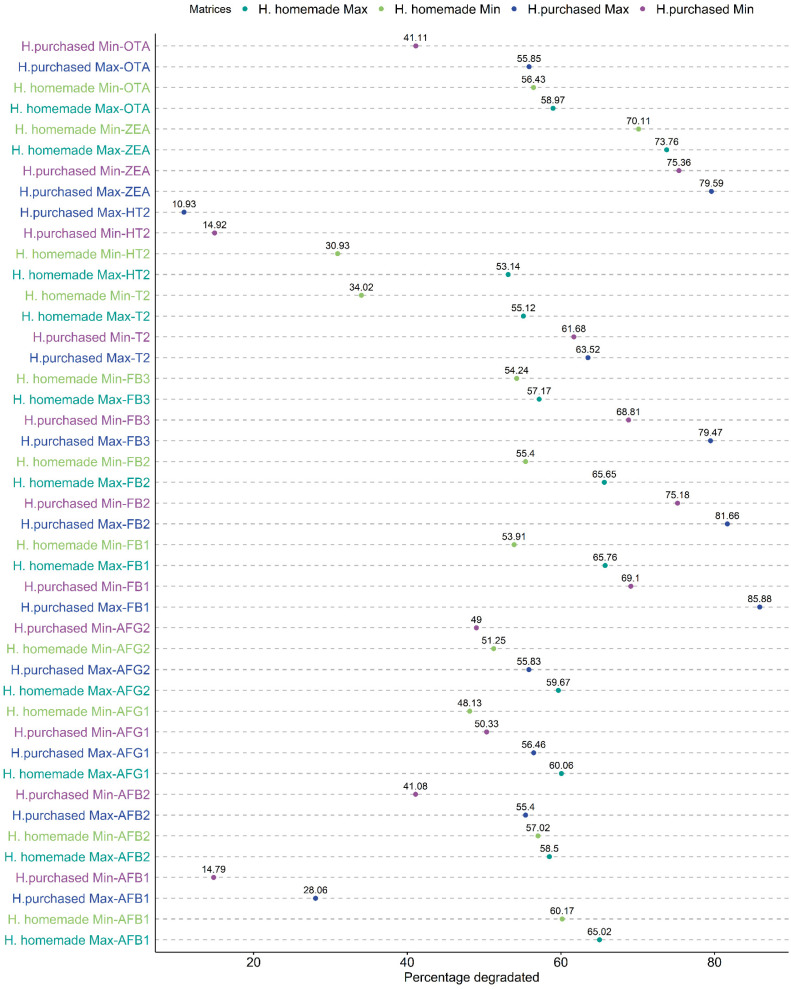
Degradation expressed as percentage losses of mycotoxins analyzed. “Min” refers to 800 w for 60 s, while “Max” is 800 w for 90 s.

In this study, a total of 14 categories of samples were analyzed. Controls (Max–Min), Carrots (Max–Min), Zucchini (Max–Min), Potatoes (Max–Min), Soybeans (Max–Min), H. homemade (Max–Min), and H. purchased (Max–Min). The differences between the matrices with the same conditions are shown in Figure 1, Figure 2 and Figure 3. The mean values of mycotoxins found are shown in Table 1 and multivariate analysis in Figure 4, while the impact of degradation of AFs (AFB1, AFB2, AFG1, AFG2), FBs (FB1, FB2, FB3), OTA, T2, HT2, and ZEA in soy burgers is shown in Figure 5. In all the conditions, with the exclusion of controls, added water evaporated completely. Temperatures recorded ranged from a minimum of 90 °C to a maximum of 98.3 °C in food matrices and 82.5–80.3 °C in control (Table 1).

#### 3.1.1. Aflatoxins

In the analysis of aflatoxins (AFs), zucchini under the “Min” condition demonstrated a high persistence of AFs, with AFB1 reaching 1.42 ± 0.066, AFB2 at 0.344 ± 0.021, and AFG1 at 1.50 ± 0.023. Carrots, also under the “Min” condition, exhibited notable levels of AFB1 at 1.54 ± 0.04 and AFG1 at 1.45 ± 0.105. Potatoes under the “Max” condition exhibited the lowest AFB1 level at 0.324 ± 0.023 and a low AFG1 level at 1.13 ± 0.048, suggesting a higher degree of AF degradation. In the case of hamburgers, homemade variants (H. homemade) under the “Max” condition showed a reduced level of AFB1 at 0.56 ± 0.064 compared to the purchased ones (H. purchased), which showed 1.15 ± 0.018 of AFB1. Regarding differences between cooking conditions, the different treatments showed significant differences for AFB1 and AFG1 (four out of six matrices) while AFB2 and AFG2 were the least sensitive to prolonged cooking times (one out of six matrices).

#### 3.1.2. Fumonisins

Zucchini under the “Min” condition emerged with the highest FB1 concentration at 373.85 ± 3.392. Carrots also showed high FB1 levels under “Min”, peaking at 363.55 ± 45.391. Potatoes under “Max” displayed considerably lower FB1 levels at 194.42 ± 1.141, suggesting greater FB1 degradation. Hamburgers, both purchased (H. purchased) and homemade (H. homemade), exhibited lower FB1 concentrations under “Max”, with purchased hamburgers at 56.47 ± 0.328 and homemade at 136.94 ± 2.385. This pattern was the same for FB2 and FB3, with zucchini and carrots showing higher concentrations than potatoes, yet zucchini’s FB2 and FB3 levels did not reach the high levels of FB1. Both hamburger types also showed reduced FB2 and FB3 levels, consistent with FB1 trends. FBs were sensible to the prolonged heat treatment in almost all matrices, with FB2 and FB1 different between the Min–Max conditions in 100% and 66.6% of the matrices analyzed.

#### 3.1.3. HT2 and T2

Carrots, under the “Min” condition, emerged with the highest HT2 toxin concentration, reaching 23.59 ± 1.42, suggesting a lower degradation rate of HT2 in carrots under milder cooking conditions. Zucchini and potatoes, under the “Max” condition, showed lower HT2 levels, with zucchini at 22.47 ± 0.105 and potatoes at 17.84 ± 0.276, indicating better degradation efficiency, especially in potatoes. For hamburgers, a difference in HT2 degradation was noted between purchased (H. purchased) and homemade (H. homemade) variants under “Max”. P. hamburgers had a higher HT2 concentration of 22.27 ± 1.667, while homemade hamburgers showed a lower level at 11.71 ± 0.074, indicating more effective degradation in the homemade variety. The differences between the Min–Max conditions was significant in 50% of the matrices.

Regarding T2 toxin, zucchini exhibited the highest level among the food items tested under the “Max” condition, with a concentration of 23.52 ± 1.150. Carrots followed, showing 24.98 ± 0.483 under “Min”, while potatoes had a lower concentration at 24.28 ± 0.677 under “Max”. In hamburgers, both purchased and homemade variants showed lower T2 levels compared to vegetables, with purchased hamburgers at 11.04 ± 1.510 and homemade at 10.26 ± 0.273 under “Max”. The differences between the conditions of cooking were relevant for soybeans and purchased hamburgers.

#### 3.1.4. OTA

Zucchini displayed the highest OTA concentration among the tested vegetables, peaking at 2.609 ± 0.061 under the “Min” condition, suggesting lower degradation efficiency during microwave cooking. Carrots showed moderate OTA levels under “Min”, with a peak at 2.41 ± 0.146, indicating a moderate degradation rate. Soybeans under “Max” had a peak OTA concentration of 1.485 ± 0.029, demonstrating slightly better degradation efficiency than zucchini but still retaining significant levels. Potatoes showed the lowest OTA level at 1.014 ± 0.007 under “Max”, suggesting a higher degradation efficiency. For hamburgers, both purchased and homemade varieties under “Max” exhibited lower OTA levels than vegetables. P. hamburgers had a peak OTA level of 1.094 ± 0.066, significantly lower than most vegetable samples. H. hamburgers showed a slightly higher OTA concentration than purchased ones, reaching 1.346 ± 0.055, but still lower than the levels in vegetables. The differences between “Max” and “Min” conditions were statistically significant in all the matrices except for both hamburgers.

#### 3.1.5. ZEA

Zucchini, under the “Min” condition, showed the highest concentration of ZEA among the tested food items, reaching a maximum of 51.862 ± 0.838. This suggests that zucchini is more prone to retaining high ZEA levels during microwave cooking. Following zucchini, carrots exhibited a notable ZEA level under “Min”, peaking at 33.734 ± 0.758, indicating a significant presence of ZEA post-cooking. Potatoes, however, demonstrated a lower ZEA level under the “Max” condition, with a maximum of 16.988 ± 0.495, suggesting a higher degradation rate of ZEA. In hamburgers, both purchased (H. purchased) and homemade (H. homemade) variants under “Max” showed lower ZEA concentrations compared to vegetables. P. hamburgers had a maximum ZEA level of 15.31 ± 0.918, significantly lower than that in vegetables. H. hamburgers also displayed lower ZEA levels, with a concentration of 19.682 ± 0.137. This pattern indicates that hamburgers, regardless of being purchased or homemade, tend to retain lower levels of ZEA when microwaved compared to vegetable samples. In carrots, zucchini, and potatoes there were statistically significant differences between Min and Max conditions.

### 3.2. Statistical Analysis

The results of the Kruskal–Wallis H test for analyzing the differences in mycotoxin levels across different food matrices showed that all mycotoxins were different between matrices (*p* < 0.05 for all the mycotoxins). Regarding hamburgers, the Wilcoxon rank-sum test results indicate that there are not significant differences in the levels of several mycotoxins between homemade and purchased hamburgers. Specifically, no significant differences were observed for mycotoxins cooked in the same conditions between purchased and homemade hamburgers (*p* ≥ 0.100). This finding indicates that the type of hamburger (homemade vs. purchased) does not significantly influence the mycotoxin content under the specific cooking conditions tested.

However, it is worth noting that the percentages of mycotoxins lost due to the cooking conditions (Figure 5) were different for all the mycotoxins tested.

Regarding PCA, PC1 opposes individuals such as “Carrots-Min” and “Zucchini-Min” (to the right of the graph, characterized by a strongly positive coordinate on the axis) to samples such as “H. purchased-Max”–“Soybeans-Max”, (to the left of the graph, characterized by a strongly negative coordinate on the axis). The group including “Zucchini-Min” had high values for AFB2, ZEA, AFB1, OTA, T2, AFG1, and FB1. “Carrots-Min” is characterized by high values for FB3 and FB2. “H. purchased-Max” has values that do not differ significantly from the mean. “Soybeans-Max” has low HT2, AFB1, AFB2, and T2 values. Regarding PC2, it opposes samples such as “H. purchased-Max” and “H. purchased-Min” (at the top of the graph, characterized by a strongly positive coordinate on the axis) to individuals such as “Carrots-Min” and “Soybeans-Min” (at the bottom of the graph, characterized by a strongly negative coordinate on the axis). The group including “H. purchased-Max” and “H. purchased-Min” has values that do not differ significantly from the mean. The group including “Soybeans-Min” has low HT2, AFB1, AFB2, and T2 values. The group including “Carrots-Min” has high values for FB3 and FB2. H. homemade, Soybeans, and H. purchased are in the same part of PC1. Therefore, they are characterized by a similar pathway in the degradation of mycotoxins.

## 4. Discussion

### 4.1. Mycotoxin Degradation Difference for Cooking Conditions

There are different data about the degradation of mycotoxins due to heat treatment in various matrices [27,35]. Industries use cooking methods such as boiling to reduce AF contents in cereals. For example, a significant reduction of AFB1 (around 94%) in maize was achieved by tortilla industries after nixtamalization, which involves boiling under alkaline conditions [36]. Other methods include roasting, bakery processing, ozone treatment, and UV irradiation [35]. Each method can mitigate mycotoxin content, influenced by the food matrix, temperature, and treatment duration [26]. Moisture content seems to be one of the key factors that can enhance the degradation of mycotoxins during food processing [25,37,38]. Our analysis revealed a general trend where extended cooking times led to reductions in most mycotoxins, depending on the category. In terms of *Aspergillus* mycotoxins (AFB1, AFB2, AFG1, and AFG2), under the Max cooking condition, significant reductions in AFB1 and AFG1 were observed across most matrices, while AFB2 and AFG2 proved more heat stable. This finding contrasts with the literature, which suggests that extremely prolonged cooking can enhance mycotoxin degradation [25,39,40]. The addition of water in the Falcon tube increases the degradation of AFs, affecting the stability of the lactone ring characteristic of AFs [41]. In fact, water in microwave heating can participate in the degradation of AFs and not act only as a solvent. The degradation does not depend only on heat treatment and microwaves can also participate [42,43,44,45].

For *Fusarium* mycotoxins (FB1, FB2, and FB3), significant reductions were observed across all matrices when subjected to the extended cooking time of 90 s. The additional 30 s of cooking at 800 W significantly reduced these mycotoxins in nearly all tested matrices, indicating their thermolability, as reported in the literature [46,47]. Interestingly, HT2 shows a slight decrease in all matrices except for homemade hamburgers and potatoes where degradation was more pronounced. The variability in response to microwave cooking among different mycotoxins and matrices aligns with literature reports of T2 and HT2 [48,49]. Specifically, the presence of water can enhance the degradation of T2 [25,50]. Schmidt et al. (2017) demonstrated that 35% moisture in oat flour can significantly increase T2 degradation during processing by over 50% [51]. OTA and ZEA generally show a decrease under extended cooking, with statistical differences in most matrices, except for both hamburgers and soybeans, where the variations were not statistically significant. This is contradictory to literature reports where a low percentage of OTA loss was observed after various cooking techniques like frying, boiling, and microwaving [37,40,41,52]. The same applies to ZEA, a heat-resistant mycotoxin that withstands common cooking methods and is unaffected by moisture [53]. The reduced degradation of mycotoxins in solution compared to food matrices can likely be attributed to the non-evaporation of water and its ability to absorb microwave energy, shielding the mycotoxins [54]. Water, with its high specific heat capacity, acts as a thermal buffer and does not easily reach boiling point. In contrast, in food matrices where water evaporates, mycotoxins are directly exposed to microwave energy, accelerating their degradation [54]. The rapid evaporation of water during microwave cooking leads to the direct exposure of mycotoxins on matrix surfaces to microwaves.

### 4.2. Mycotoxin Degradation in Hamburgers

Microwave heating’s role in reducing aflatoxins has been studied in cereals, with a maximum reduction of 32% after 10 min at 900 W [39]. In chicken breast, characterized by higher moisture levels, the reduction of AFs post-microwave cooking was greater, exceeding 50% (50.7–78.6% AFB1, 46.2–84.6% AFG2) [25]. In our study, AF degradation was higher in H. homemade (AFB1 65%, AFB2 59.17%, AFG1–AFG2 60%) than in H. purchased (AFB1 28.13%, AFB2 55.83%, AFG1 56.43%, AFG2 56.67%), possibly due to the homemade hamburger’s different composition and texture, which increased the exposure of AFs to heated water within the matrix. However, it is important to note that, despite the differences in loss percentages, the statistical analysis showed no significant difference in mycotoxins between homemade and purchased hamburgers under each condition.

FBs showed greater degradation in purchased hamburgers compared to homemade ones. The degradation reaches a peak of 85.88% (FB1) in purchased hamburgers and cooked at maximum conditions, while the minimum reduction was observed in homemade hamburgers cooked at minimum conditions (53.91%). These percentages are in accordance with the literature where a reduction of up to 50% was observed in chips (frying and extrusion) [28] and in chicken breast cooked in a microwave (42%) [25]. However, FBs can bind food matrix components such as protein and these compounds are called “hidden fumonisins” and cannot be detected easily [55]. The greater thermolability of FBs is also evident, as both cooking conditions consistently resulted in significant variations in their quantities within each matrix, with the exception of FB1 in soybeans and FB3 in carrots and hamburgers (gray in Table 1, Figure 2).

T2 and HT2 exemplified remarkable stability post-heat treatment, aligning with existing scholarly discourse that identifies these mycotoxins as resilient to thermal processes [25]. The attrition in HT2 levels varied, ranging from 10.93% (Max, H. purchased) to 53.14% (Max, H. homemade), indicating a differential thermal susceptibility. T2 exhibited less resistance to heat across all matrices relative to HT2. These findings are corroborated by the research of Kuchenbuch et al. (2018), who observed similar patterns in heat-treated biscuits and crunchy muesli in the oven [50].

In the case of ZEA, the observed degradation was pronounced, fluctuating between a minimum of 53.14% and a maximum of 79.59%. Notably, the degradation rates were consistently higher in H. purchased samples. This contrasts with existing literature that reports lower degradation rates for ZEA, typically characterizing it as a heat-resistant mycotoxin. In particular, ZEA degradation percentages reported were less than 20% in microwaved chicken breast [25] and fried potatoes [40].

The degradation of OTA exhibited variability between homemade hamburgers (58.97% Max, 56.43% Min) and purchased hamburgers (55.85% Max, 41.11% Min), with a marked increase in degradation across all conditions in the purchased variant. This observation stands in contrast to prior studies suggesting that increased moisture content may impede OTA degradation [56]. Investigations into microwave cooking of chicken breast revealed OTA degradation percentages of approximately 39.1% and 25.1% at 700 W for 15 min [25]. Our findings seem more congruent with studies indicating a substantial 84% degradation of OTA in beans subjected to pressure cooking in water [57,58], possibly attributable to a semi-open-cap environment that simulates steam cooking due to the presence of 5 mL of water during the process.

These results underscore the significance of matrix-specific characteristics and food composition in influencing the thermal stability of mycotoxins. Each matrix demonstrated distinct mycotoxin profiles, necessitating further exploration of diverse matrices to elucidate the extent of mycotoxin contamination and its modulation during food processing. The principal component analysis (PCA) furnished a nuanced perspective on the mycotoxin profiles across various food matrices and preparation methodologies. The observed distinct clustering of certain sample groups suggests unique patterns of mycotoxin contamination and degradation. For instance, soybeans and hamburgers, both purchased and homemade, aligned along the same trajectory in PC1, indicative of similar mycotoxin degradation patterns.

## 5. Study Limitations

The study conducted has several limitations. First, the method is not fully validated and parameters were not calculated for each matrix. Considering the matrix effect that can enhance or suppress the signal, it is important to fully validate a method that encompasses the analyses of PBMAs, ingredients included. The study showed that each matrix is different, therefore other types of products must be studied (such as veggie sausages and veggie meatballs). Considering the plethora of ingredients that can be used for these products (such as lupins), the matrices analyzed are few. Furthermore, spiked samples cannot be indicative of naturally contaminated samples. Therefore, it would be beneficial to confirm these findings with real samples. The mechanism of degradation due to temperature or microwaves still unclear.

## 6. Conclusions

This study augments the current understanding of mycotoxin behavior in food matrices under cooking conditions, particularly focusing on matrices not yet regulated by European regulation. The findings, highlighting the degradation of certain mycotoxins such as OTA, T2, and HT2 during microwave cooking, contribute to the existing literature on the heat-resistant nature of these compounds. Adding water during microwave cooking can help to reduce AFs to a high level. There is a pressing need for additional research to facilitate a more nuanced comparison of food matrices and their degradation patterns, given the wide array of products available as PBMAs.

## Figures and Tables

**Table 1:** Mycotoxin (expressed as mean ± standard deviation μg/kg). The concentrations that were statistically different between “Min” and “Max” conditions (*p* < 0.05) are shown in gray. “Control” refers to the aqueous standard solution of mycotoxins.

Analytes	Type	Carrots	Zucchini	Potatoes	Soybeans	H. purchased	H. homemade	Control	Spiked
AFB1	Max	1.41 ± 0.06	1.264 ± 0.147	0.324 ± 0.023	0.584 ± 0.026	1.151 ± 0.018	0.56 ± 0.064	1.462 ± 0.066	1.6
	Min	1.542 ± 0.044	1.422 ± 0.066	0.514 ± 0.06	0.676 ± 0.021	1.363 ± 0.022	0.637 ± 0.072	1.565 ± 0.045	
AFB2	Max	0.177 ± 0.07	0.218 ± 0.026	0.112 ± 0.083	0.091 ± 0.048	0.178 ± 0.04	0.166 ± 0.022	0.36 ± 0.022	0.4
	Min	0.211 ± 0.098	0.344 ± 0.021	0.184 ± 0.047	0.18 ± 0.027	0.236 ± 0.05	0.172 ± 0.037	0.371 ± 0.026	
AFG1	Max	1.35 ± 0.14	1.287 ± 0.031	1.13 ± 0.048	0.591 ± 0.032	0.697 ± 0.098	0.639 ± 0.045	1.417 ± 0.032	1.6
	Min	1.448 ± 0.105	1.503 ± 0.023	1.286 ± 0.051	0.675 ± 0.035	0.795 ± 0.031	0.83 ± 0.017	1.521 ± 0.062	
AFG2	Max	0.348 ± 0.02	0.288 ± 0.049	0.286 ± 0.056	0.115 ± 0.037	0.177 ± 0.022	0.161 ± 0.003	0.347 ± 0.043	0.4
	Min	0.35 ± 0.041	0.359 ± 0.036	0.313 ± 0.05	0.213 ± 0.045	0.204 ± 0.013	0.195 ± 0.044	0.382 ± 0.014	
FB1	Max	283.164 ± 38.94	209.443 ± 1.562	194.419 ± 1.141	124.545 ± 0.263	56.474 ± 0.328	136.944 ± 2.385	338.83 ± 9.794	400
	Min	363.552 ± 45.391	373.848 ± 3.392	356.692 ± 3.332	298.861 ± 3.558	123.584 ± 2.143	184.36 ± 1.335	367.165 ± 12.819	
FB2	Max	210.288 ± 5.34	189.176 ± 6.928	152.419 ± 0.305	127.898 ± 2.938	73.36 ± 1.599	137.405 ± 1.79	264.365 ± 23.388	400
	Min	285.901 ± 5.12	206.331 ± 4.959	201.751 ± 1.213	181.773 ± 1.063	99.296 ± 0.568	178.417 ± 1.959	283.163 ± 16.332	
FB3	Max	336.882 ± 6.99	225.779 ± 6.124	225.165 ± 2.141	140.381 ± 1.332	82.125 ± 0.876	171.33 ± 2.122	349.522 ± 20.247	400
	Min	368.691 ± 36.548	282.031 ± 4.314	251.728 ± 2.627	229.382 ± 5.42	124.756 ± 0.737	183.055 ± 8.718	375.667 ± 23.865	
HT2	Max	21.635 ± 2.57	22.467 ± 0.105	17.843 ± 0.276	6.9 ± 0.061	22.268 ± 1.667	11.715 ± 0.074	21.834 ± 1.298	25
	Min	23.595 ± 1.421	24.741 ± 0.092	20.258 ± 0.121	8.004 ± 0.135	21.27 ± 0.279	17.268 ± 0.151	24.646 ± 0.608	
T2	Max	24.356 ± 0.63	23.518 ± 1.15	24.277 ± 0.677	9.555 ± 0.078	11.037 ± 1.51	10.257 ± 0.273	22.607 ± 0.529	25
	Min	24.644 ± 0.247	23.885 ± 0.543	23.611 ± 0.941	11.631 ± 0.092	14.721 ± 0.295	10.893 ± 0.611	24.296 ± 1.171	
OTA	Max	1.473 ± 0.06	2.172 ± 0.008	1.014 ± 0.007	1.485 ± 0.029	1.094 ± 0.066	1.346 ± 0.055	2.384 ± 0.09	3
	Min	2.41 ± 0.146	2.609 ± 0.061	1.402 ± 0.003	2.602 ± 0.032	1.15 ± 0.013	1.979 ± 0.075	2.598 ± 0.115	
ZEA	Max	28.938 ± 1.39	47.744 ± 0.164	16.988 ± 0.495	37.978 ± 0.335	15.31 ± 0.918	19.682 ± 0.137	47.317 ± 4.679	75
	Min	33.734 ± 0.758	51.862 ± 0.838	23.745 ± 0.138	40.247 ± 0.349	18.482 ± 1.631	22.415 ± 1.943	50.767 ± 7.217	
Temp.	Max	93.5 ° C	95.2 °C	97.6 °C	92 °C	93.2 °C	91 °C	82.5 °C	
	Min	94 °C	95.3 °C	98.3 °C	94.3 °C	91.8 °C	90 °C	80.3 °C	

## Data Availability

The data presented in this study are available on request from the corresponding author. The data are not publicly available due to part of an ongoing study.
